# Correction

**DOI:** 10.1080/27694127.2026.2727268

**Published:** 2026-09-02

**Article title**: Glucose concentration of neuronal media formulations influences PINK1-dependent mitophagy in human iNeurons.

**Authors**: O’Callaghan B., Melandri D., Soltic D., Cosker K., Soutar M. P. M., Evans J.R., Lucas-Clarke H., Robin J., Gandhi S., Birsa N., Arber C., Wray S., & Plun-Favreau H.

**Journal**: KAUO: Autophagy Reports.

**Bibliomerics**: Volume 05, Number 01, pages 01 - 19.

**DOI**: https://doi.org/10.1080/27694127.2026.2685472.

Initially, the history dates in the article were published with an error, which has now been corrected and republished.

The corrected history dates are:

Received: 04 Sep 2025.

Accepted: 03 June 2026.

