# Supplementary material for: Glucose concentration of neuronal media formulations influences PINK1-dependent mitophagy in human iNeurons
Source: Autophagy Rep. 2026 Jun 12;5(1):2685472. doi: 10.1080/27694127.2026.2685472 (PMC13274156; doi:10.1080/27694127.2026.2685472)
Supplement: Supplemental Material [file KAUO_A_2685472_SM0446.docx]

**Extended Data Figure 1 – The intensity of pUb(Ser65) within MAP2^+^ iNeurons is reduced when cultured in BrainPhys.** (**A**) Representative confocal maximum intensity projections of pUb(Ser65) immunofluorescence (green) following treatment of d24 N2B27 and BrainPhys iNeurons with DMSO or 1 µM O/A. Insets show the Hoechst 33342 counterstained nuclei (blue) and MAP2 immunofluorescence (magenta) for the same field of view. Scale bars = 50 µm. (**F**) Quantification of mean pUb(Ser65) intensity within MAP2^+^ regions in (**E**) (n=5 inductions, 6x 323 µm x 323 µm fields of view per well, 4-6 experimental wells per condition, two-way ANOVA with Šidák *post hoc* correction).
Data are shown as the mean with error bars ± SD from biological replicates, with normalised values for each biological replicate shown as individual markers.

**Extended Data Figure 2 – Cortical Neurons differentiated through dual SMADi show impairments in PINK1-dependent mitophagy initiation when cultured in BrainPhys.** (**A**) Representative immunoblots of d76 cortical neurons cultured in N2B27 vs BrainPhys medium for 3 weeks prior to collection, and treated with 1 µM O/A over a 24h time-course. (**B**) Quantification of pUb(Ser65) from (**A**) (n=3 inductions from each from a separate control hPSC line/genotype, two-way ANOVA with Šidák *post hoc* correction).
Data are shown as the mean with error bars ± SD from biological replicates, with normalised values for each biological replicate shown as individual markers.

**Extended Data Figure 3 – Reductions in O/A induced FL-PINK1 protein availability in BrainPhys iNeurons do not appear to be linked with enhanced cleavage of PINK1 (ΔPINK1) and its subsequent degradation.** (**A**) Representative immunoblots of d24 iNeurons cultured in N2B27 vs BrainPhys medium and treated with 1 µM O/A, 10 µM MG132 or co-application of O/A + MG132 for 9h. (**B-D**) Quantification of full-length PINK1 (FL-PINK1) (**B**), PARL-cleaved PINK1 (ΔPINK1) (**C**) and calculation of the FL-PINK1:ΔPINK1 ratio from (**A**) (n=4 inductions, two-way ANOVA with Šidák *post hoc* correction).
Data are shown as the mean with error bars ± SD from biological replicates, with normalised values for each biological replicate shown as individual markers.

**Extended Data Figure 4 – BrainPhys iNeurons have a more depolarised basal mitochondrial membrane potential and show comparable levels of mitochondrial membrane potential following application of a range of different mitochondrial toxins and doses.** (**A**) Representative confocal maximum intensity projections of d24 N2B27 and BrainPhys iNeurons stained with 25 nM TMRM, and pretreated for 2h with DMSO or various mitochondrial toxins (number indicates [toxin] in µM; ROT = rotenone, O = Oligomycin). Insets show the YPet^+^ mitochondria used for defining TMRM measurement areas for the same field of view. Scale bars = 50 µm. (**B**) Quantification of mean TMRM intensity within YPet^+^ mitochondrial regions in (**A**) (n=3 inductions, 5x 205 µm x 205 µm fields of view per well, 3 experimental wells per condition, two-way ANOVA with Šidák *post hoc* correction).
Data are shown as the mean with error bars ± SD from biological replicates, with normalised values for each biological replicate shown as individual markers.

**Extended Data Figure 5 – BrainPhys iNeurons show impairments in pUb(Ser65) deposition and PINK1 accumulation following application of a range of different mitochondrial toxins and doses.** (**A**) Representative immunoblots of d24 iNeurons cultured in N2B27 vs BrainPhys medium and treated for 9h with DMSO or various mitochondrial toxins (number indicates [toxin] in mM; ROT = rotenone, O = Oligomycin). (**B-C**) Quantification of pUb(Ser65) (**B**) and PINK1 (**C**) from (**A**) (n=3 inductions, two-way ANOVA with Šidák *post hoc* correction).
Data are shown as the mean with error bars ± SD from biological replicates, with normalised values for each biological replicate shown as individual markers.

**Extended Data Figure 6 – Differences in PINK1 protein availability between N2B27 and BrainPhys iNeurons is at least partly due to differences in glucose availability.** (**A**) Representative immunoblots of d24 iNeurons cultured in N2B27 vs BrainPhys medium with low 2.5mM glucose or high 21.25mM glucose and treated with 1 µM O/A or 10 µM MG132 for 9h. (**B-C**) Quantification of full-length PINK1 (FL-PINK1) (**B**) and PARL-cleaved PINK1 (ΔPINK1) (**C**) from (**A**) (n=4 inductions, two-way ANOVA with Šidák *post hoc* correction).
Data are shown as the mean with error bars ± SD from biological replicates, with normalised values for each biological replicate shown as individual markers.
