# Supplementary material for: Glucose concentration of neuronal media formulations influences PINK1-dependent mitophagy in human iNeurons
Source: Autophagy Rep. 2026 Jun 12;5(1):2685472. doi: 10.1080/27694127.2026.2685472 (PMC13274156; doi:10.1080/27694127.2026.2685472)
Supplement: Supplemental Material [file KAUO_A_2685472_SM0443.pdf]

Extended_Data_Figure_1 2
Extended_Data_Figure_2 3
Extended_Data_Figure_3 4
Extended_Data_Figure_4 5
Extended_Data_Figure_5 6
Extended_Data_Figure_6 7

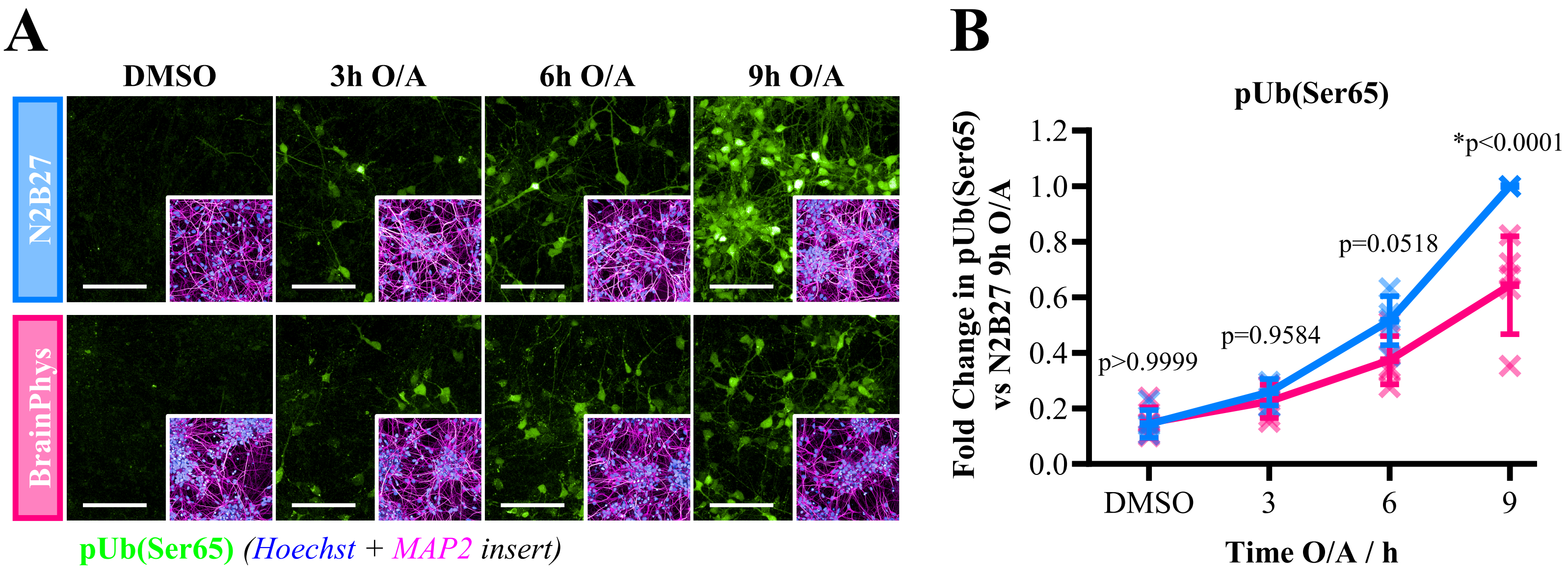

A
DMSO
3h O/A
6h O/A
9h O/A
N2B27
BrainPhys
pUb(Ser65) (Hoechst + MAP2 insert)
B
pUb(Ser65)
Fold Change in pUb(Ser65) vs N2B27 9h O/A
1.2
1.0
0.8
0.6
0.4
0.2
0.0
*p<0.0001
p=0.0518
p=0.9584
p>0.9999
DMSO
3
6
9
Time O/A / h

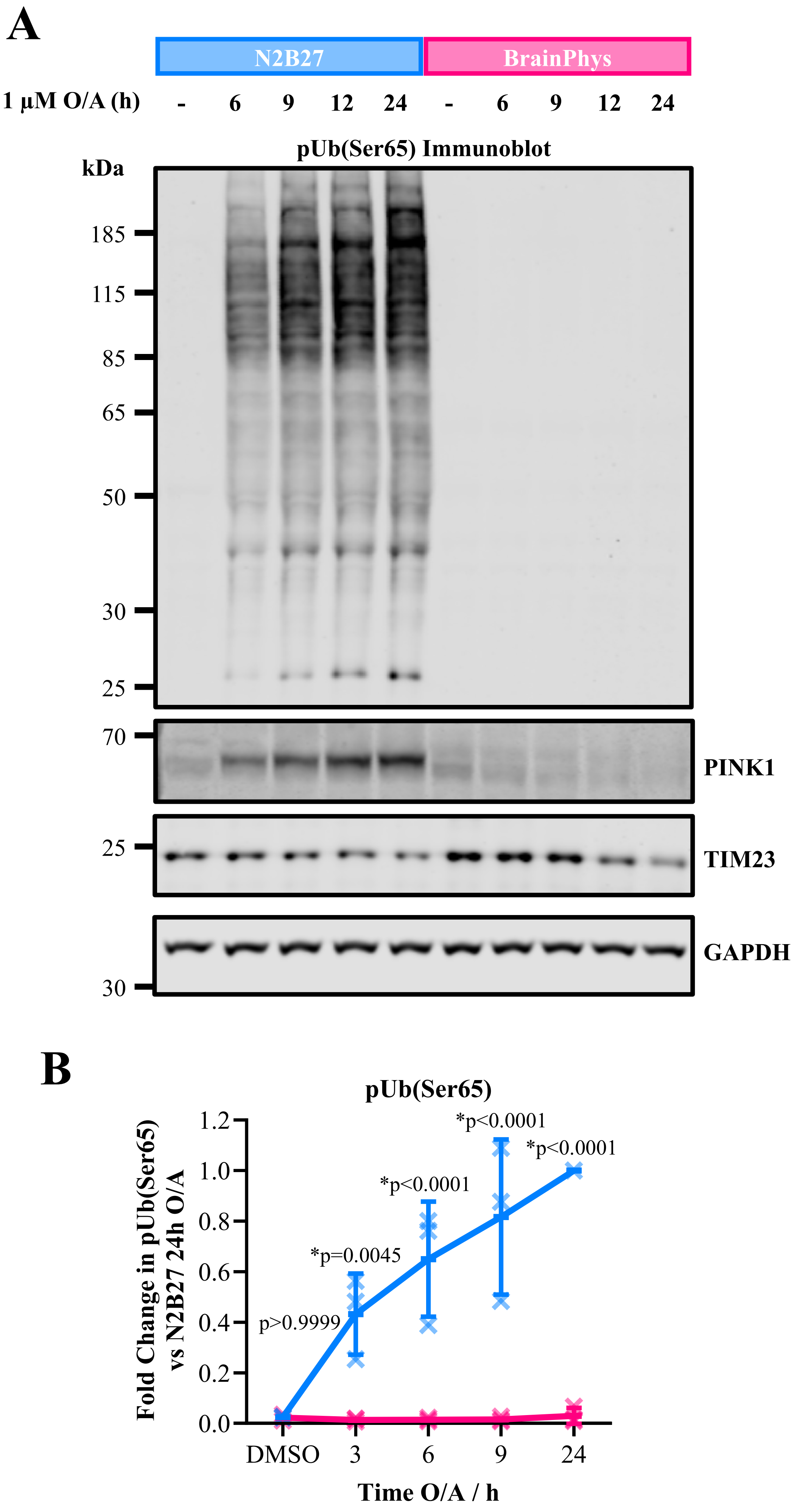
A
N2B27
BrainPhys
1 µM O/A (h)
- 6 9 12 24 - 6 9 12 24
pUb(Ser65) Immunoblot
kDa
185
115
85
65
50
30
25
70
PINK1
25
TIM23
GAPDH
30
B
pUb(Ser65)
Fold Change in pUb(Ser65) vs N2B27 24h O/A
1.2
1.0
0.8
0.6
0.4
0.2
0.0
*p<0.0001
*p<0.0001
*p<0.0001
*p=0.0045
p>0.9999
DMSO
3
6
9
24
Time O/A / h

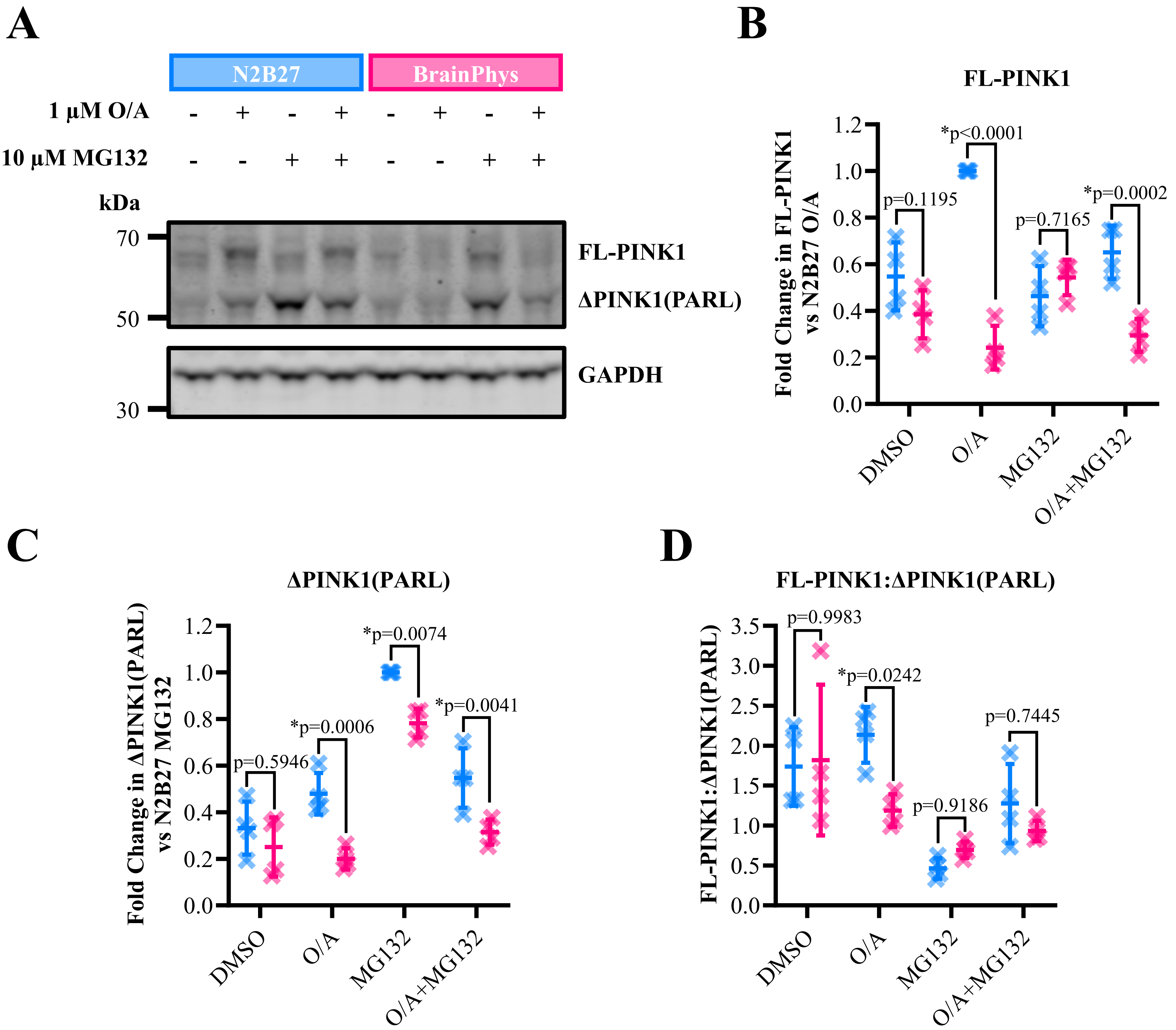
A
N2B27
BrainPhys
1 µM O/A
10 µM MG132
kDa
70
50
30
FL-PINK1
ΔPINK1(PARL)
GAPDH
B
FL-PINK1
Fold Change in FL-PINK1 vs N2B27 O/A
p=0.1195
*p<0.0001
p=0.7165
*p=0.0002
DMSO
O/A
MG132
O/A+MG132
C
ΔPINK1(PARL)
Fold Change in ΔPINK1(PARL) vs N2B27 MG132
p=0.5946
*p=0.0006
*p=0.0074
*p=0.0041
DMSO
O/A
MG132
O/A+MG132
D
FL-PINK1:ΔPINK1(PARL)
FL-PINK1:ΔPINK1(PARL)
p=0.9983
*p=0.0242
p=0.9186
p=0.7445
DMSO
O/A
MG132
O/A+MG132

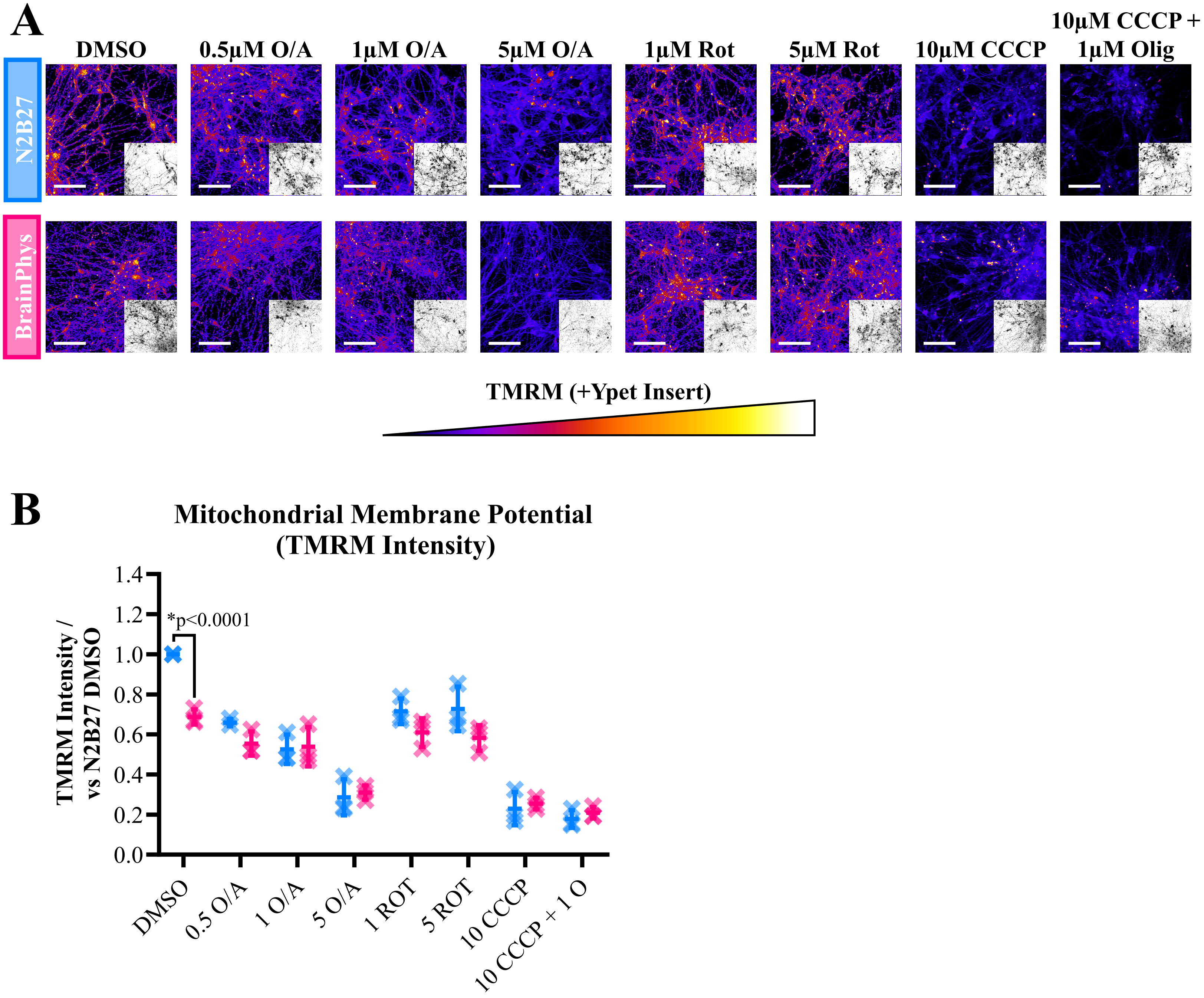
A
DMSO
0.5µM O/A
1µM O/A
5µM O/A
1µM Rot
5µM Rot
10µM CCCP
10µM CCCP +
1µM Olig
N2B27
BrainPhys
TMRM (+Ypet Insert)
B
Mitochondrial Membrane Potential
(TMRM Intensity)
TMRM Intensity /
vs N2B27 DMSO
1.4
1.2
1.0
0.8
0.6
0.4
0.2
0.0
*p<0.0001
DMSO
0.5 O/A
1 O/A
5 O/A
1 ROT
5 ROT
10 CCCP
10 CCCP + 1 O

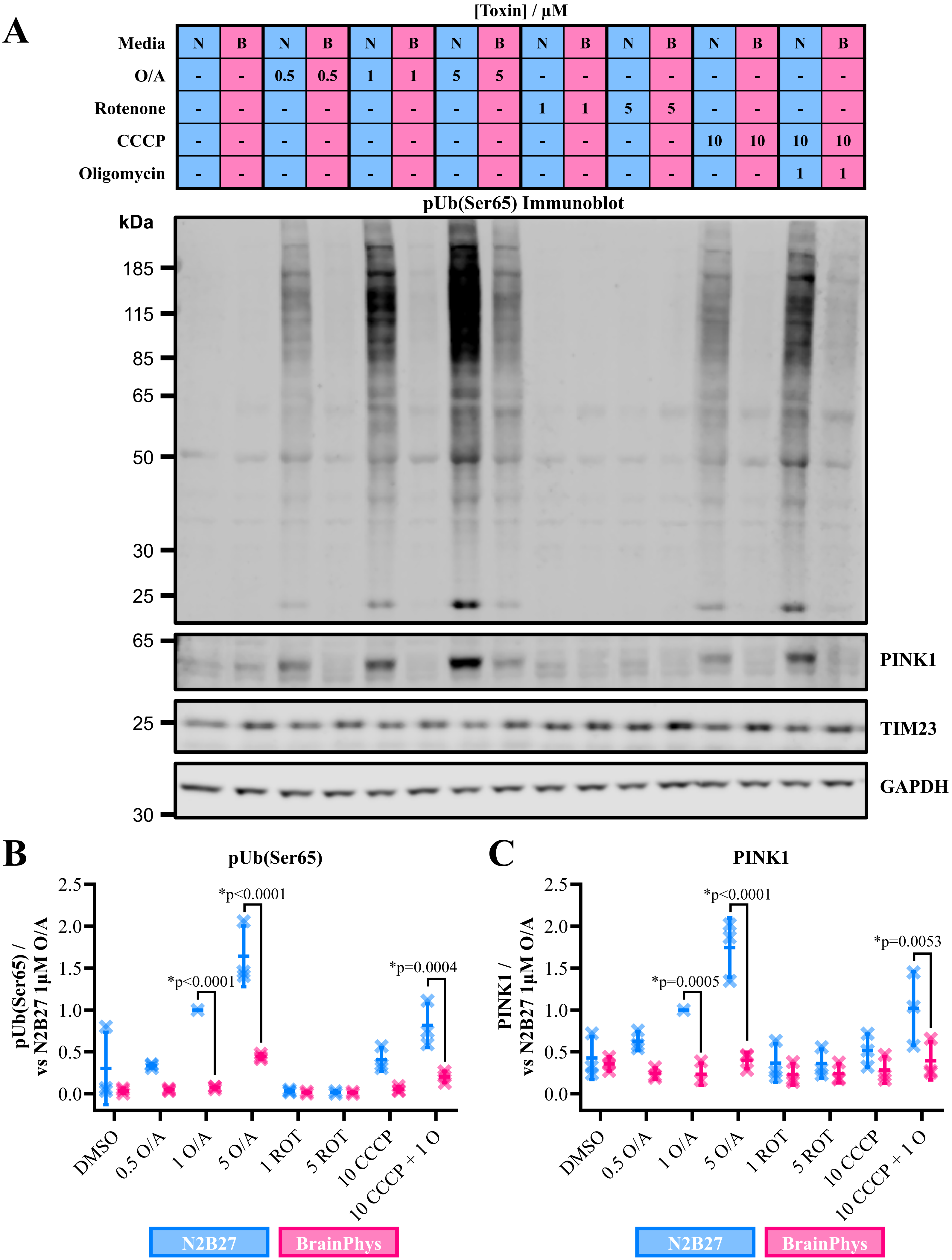

A
[Toxin] / µM
Media
O/A
Rotenone
CCCP
Oligomycin
N
B
0.5
1
5
10
pUb(Ser65) Immunoblot
kDa
185
115
85
65
50
30
25
PINK1
TIM23
GAPDH
B
pUb(Ser65)
pUb(Ser65) / vs N2B27 1µM O/A
*p<0.0001
*p<0.0001
*p=0.0004
C
PINK1
PINK1 / vs N2B27 1µM O/A
*p=0.0005
*p<0.0001
*p=0.0053
DMSO
0.5 O/A
1 O/A
5 O/A
1 ROT
5 ROT
10 CCCP
10 CCCP + 1 O
N2B27
BrainPhys

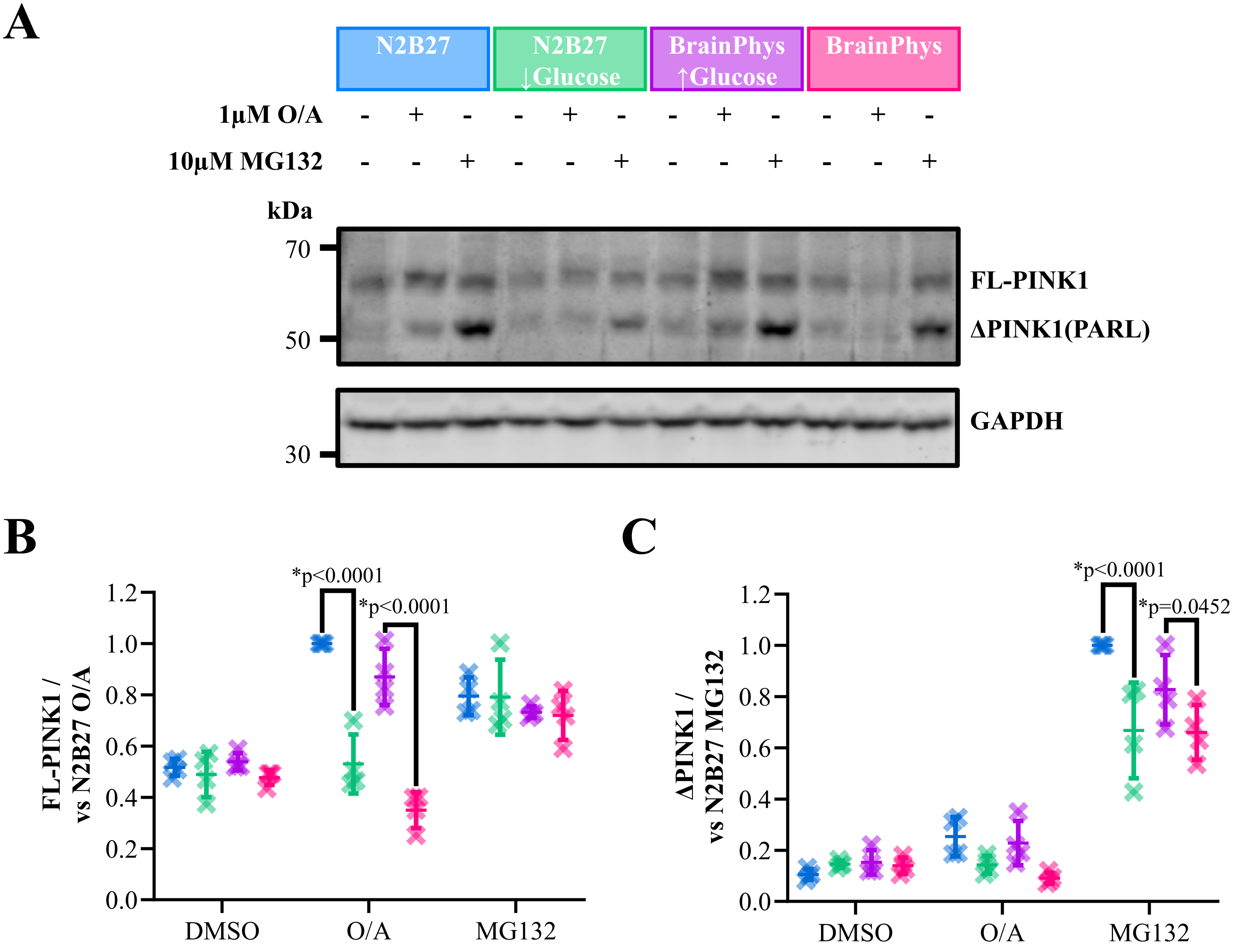

A
N2B27
N2B27 ↓Glucose
BrainPhys ↑Glucose
BrainPhys
1µM O/A
10µM MG132
kDa
70
50
30
FL-PINK1
ΔPINK1(PARL)
GAPDH
B
*p<0.0001
*p<0.0001
FL-PINK1 / vs N2B27 O/A
1.2
1.0
0.8
0.6
0.4
0.2
0.0
DMSO
O/A
MG132
C
*p<0.0001
*p=0.0452
ΔPINK1 / vs N2B27 MG132
DMSO
O/A
MG132
